# Global epigenomic analysis indicates that Epialleles contribute to Allele-specific expression via Allele-specific histone modifications in hybrid rice

**DOI:** 10.1186/s12864-015-1454-z

**Published:** 2015-03-24

**Authors:** Zhibin Guo, Gaoyuan Song, Zhenwei Liu, Xuefeng Qu, Rong Chen, Daiming Jiang, Yunfang Sun, Chuan Liu, Yingguo Zhu, Daichang Yang

**Affiliations:** State Key Laboratory of Hybrid Rice and College of Life Sciences, Wuhan University, Luojia Hill, Wuhan, 430072, Hubei Province China

**Keywords:** Allele-specific histone modifications, Rice F1 hybrids, Epialleles, Differentially modified genes, Allele-specific expression

## Abstract

**Background:**

For heterozygous genes, alleles on the chromatin from two different parents exhibit histone modification variations known as allele-specific histone modifications (ASHMs). The regulation of allele-specific gene expression (ASE) by ASHMs has been reported in animals. However, to date, the regulation of ASE by ASHM genes remains poorly understood in higher plants.

**Results:**

We used chromatin immunoprecipitation followed by next-generation sequencing (ChIP-seq) to investigate the global ASHM profiles of trimethylation on histone H3 lysine 27 (H3K27me3) and histone H3 lysine 36 (H3K36me3) in two rice F1 hybrids. A total of 522 to 550 allele-specific H3K27me3 genes and 428 to 494 allele-specific H3K36me3 genes were detected in GL × 93-11 and GL × TQ, accounting for 11.09% and 26.13% of the total analyzed genes, respectively. The epialleles between parents were highly related to ASHMs. Further analysis indicated that 52.48% to 70.40% of the epialleles were faithfully inherited by the F1 hybrid and contributed to 33.18% to 46.55% of the ASHM genes. Importantly, 66.67% to 82.69% of monoallelic expression genes contained the H3K36me3 modification. Further studies demonstrated a significant positive correlation of ASE with allele-specific H3K36me3 but not with H3K27me3, indicating that ASHM-H3K36me3 primarily regulates ASE in this study.

**Conclusions:**

Our results demonstrate that epialleles from parents can be inherited by the F1 to produce ASHMs in the F1 hybrid. Our findings indicate that ASHM-H3K36me3, rather than H3K27me3, mainly regulates ASE in hybrid rice.

**Electronic supplementary material:**

The online version of this article (doi:10.1186/s12864-015-1454-z) contains supplementary material, which is available to authorized users.

## Background

Eukaryotic DNA is wrapped around an octamer of four core histones (H2A, H2B, H3 and H4) that form nucleosomes [1]. The epigenetic regulation of gene expression can be affected by methylation on the N-terminal tails of the histones of a gene [2,3]. The differences in epigenetic modification between parents, which are known as epialleles, can be transmitted to the next generation [4]. Epigenetic modifications can be reprogrammed during development and in response to environmental stresses [5-8]. Histone modifications have multiple functions and diversity patterns [9]. Previous studies have demonstrated that the trimethylation of histone H3 on lysine 27 (H3K27me3) represses gene expression via the specific enrichment of H3K27me3 in the gene body [10-12]. In contrast, the specific enrichment of trimethylated histone H3 on lysine 36 (H3K36me3) in the gene body activates gene expression [13-15].

Epigenetic modifications are not always identical on different homologous chromosomes in diploid organisms. To identify allelic modifications, single-nucleotide polymorphisms (SNPs) have been widely used to distinguish allele-specific gene expression and epigenetic modifications, which are known as allele-specific gene expressions (ASEs), allele-specific DNA methylations and allele-specific histone modifications (ASHMs). ASHMs and allele-specific DNA methylations have been detected in plants and animals [11,16-19]. Recent studies have demonstrated that mono-ASE genes, where only one of the alleles in hybrids is expressed, play important roles in development and stress-induced responses [20-22]. The relationship between ASE and allele-specific epigenetic modifications, including allele-specific DNA methylations and histone modifications, is supported by the allele-specific epigenetic regulation of imprinting genes [23,24]. In mice, 20 genes (21.3%) have ASE or allele-specific H3K4me3 enrichment with a negative correlation with ASHM [25]. In a rice F1 hybrid, 15 ASE genes (17.6%) correlate with ASHM in the *indica-japonica* F1 hybrid [26]. However, ASHMs in the *indica-indica* rice F1 hybrid have not been studied.

In this study, allele-specific H3K27me3 and H3K36me3 modifications were analyzed in two rice F1 hybrids, Guangluai (GL) × 93-11 and GL × Teqing (TQ), and their parents. We used chromatin immunoprecipitation followed by next-generation sequencing (ChIP-seq) to investigate the ASHM patterns of H3K27me3 and H3K36me3 in GL × 93-11 and GL × TQ. We found strong correlations between ASHM in the F1 hybrids and the epialleles from the parents. Further studies indicated that 52.48% to 70.40% of the epialleles were faithfully inherited by the F1 hybrid and contributed to 33.18% to 46.55% of the ASHM genes. Importantly, 66.67% to 82.69% of the monoallelic expression genes had the H3K36me3 modification, and ASE was strong correlated with ASHM. These results indicate that the regulatory effects of ASHM-H3K36me3 on ASE are stronger than those of H3K27me3. Our results show that H3K36me3 may play an important role in the regulation of ASE in rice F1 hybrids.

## Results

### The Frequency of ASHMs in Rice F1 Hybrids

To investigate the ASHM profiles in rice hybrids, we used three elite rice *indica* varieties, Guangluai-4 (GL), Yangdao-6 (93–11) and Teqing (TQ), and two F1 hybrids (GL × 93-11 and GL × TQ) that were generated from these three varieties. These elite varieties represent the breeding objectives at different historical breeding stages in China. The gene repression epigenetic marker H3K27me3 and the gene activation epigenetic marker H3K36me3 were chosen for this study. Sequencing depths of 36.4-43.8 million unique mapped reads (49 bp per read) for H3K27me3 and 37.3-49.8 million unique mapped reads for H3K36me3 were obtained using ChIP-seq (Additional files 1 and 2). A total of 411,553 (GL versus 93–11) and 357,765 (GL versus TQ) SNPs were available for ASHM analysis from the deep sequencing of the three varieties [27]. Of the available SNPs, 206,306 to 173,464 reads in GL × 93-11 and 156,830 to 151,529 reads in GL × TQ were available for the allelic histone modification analysis of H3K27me3 and H3K36me3 (Additional file 3).

**Figure 1:**
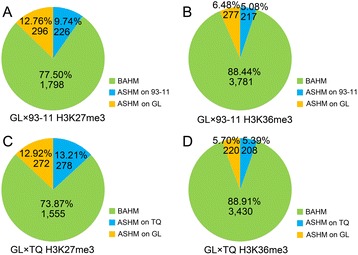
**The allelic histone modification patterns of genes in GL × 93-11 and GL × TQ. (A** **-D)**, The allelic histone modification patterns of the genes. The ASHM levels of the genes that were covered by more than nine reads were calculated. Allelic histone modification (ASHM) designates the read number of one allele with a two-fold difference from that of another allele; BAHM indicates that two alleles were equally modified.

A total of 10,647 H3K27me3-modified genes and 15,389 H3K36me3-modified genes in GL × 93-11 were detected with a threshold of read coverage defined by randomization (*P*-value < 0.001). A total of 2,320 and 4,275 genes that satisfied the criteria of more than nine SNPs in the gene body region were chosen for further allelic-specific histone modification analysis. In GL × TQ, 10,855 and 14,556 genes were modified with H3K27me3 and H3K36me3 modifications, respectively. Of these genes, 2,105 (H3K27me3-modified) and 3,858 (H3K36me3-modified) were available for ASHM analysis. We found that 296 genes (12.76%) from GL alleles and 226 genes (9.74%) from 93–11 alleles showed preferential allelic H3K27me3 modification (Figure 1A); 277 genes (6.48%) from GL alleles and 217 genes (5.08%) from 93–11 alleles showed preferential allelic H3K36me3 modification in GL × 93-11 (Figure 1B). A total of 272 genes (12.92%) from GL alleles and 278 genes from TQ alleles (13.21%) showed preferential allelic H3K27me3 modification (Figure 1C), while 220 genes (5.70%) from GL alleles and 208 genes (5.39%) from TQ alleles showed preferential allelic H3K36me3 modification (Figure 1D). Our results indicate the genome-wide frequency of ASHMs in rice F1 hybrids is much higher than that of the previous studies [11].

### Allelic histone modifications in F1 hybrids were correlated with epialleles

**Figure 2:**
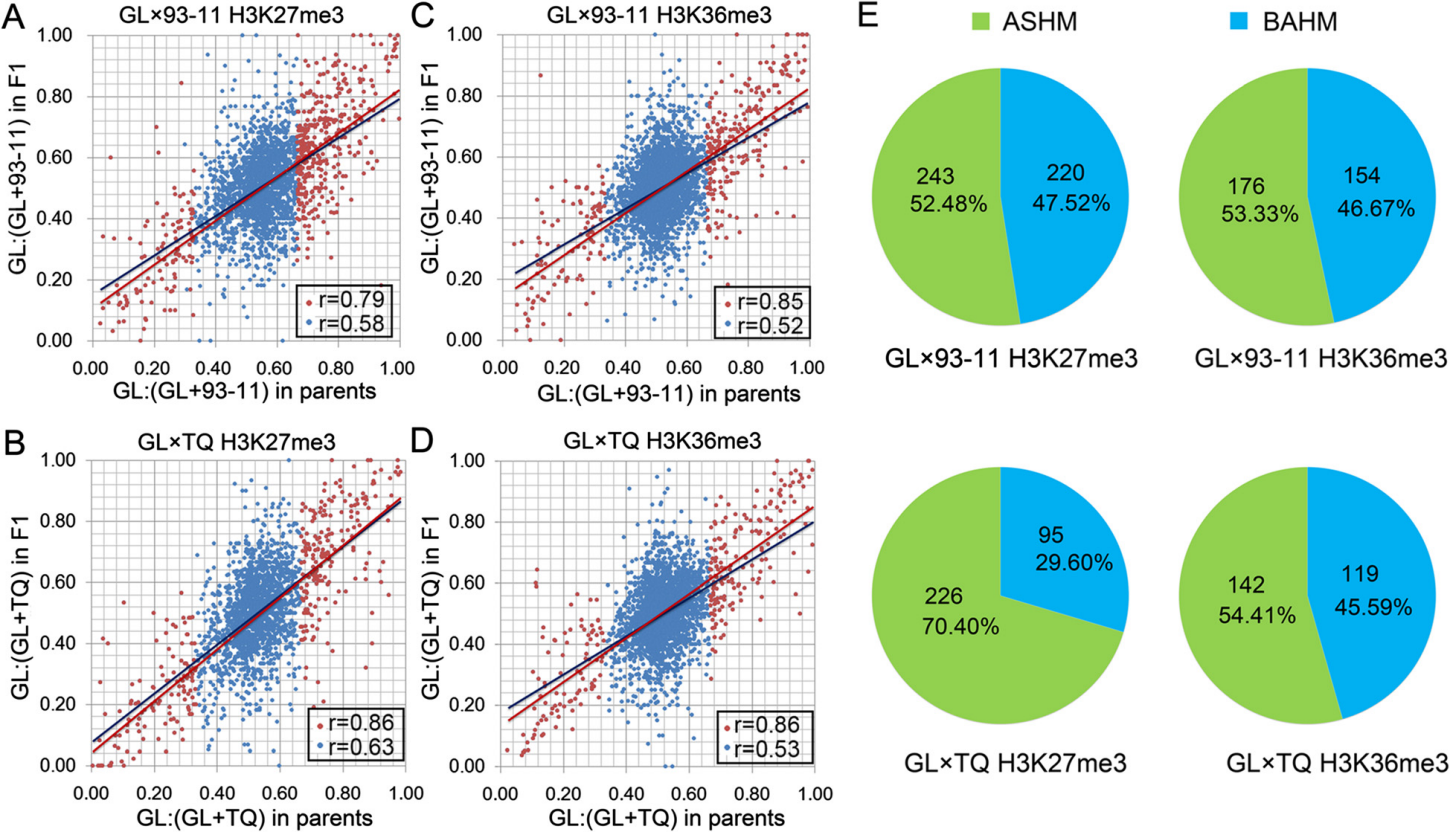
**Correlations between epialleles in the parents and ASHMs in the F1 hybrid. (A** **-D)**, The allelic histone modification difference between the hybrid and parents are presented by the ratio of the GL modification level to the total histone modification level of two alleles in the F1 hybrid or parents. The red spots indicate the genes with a different modification between parents (fold change > 2, FDR < 0.05), and the blue spot represents all of the analyzed genes. **(E)**, The ASHM patterns of the epialleles in the F1 hybrids.

Previous studies have demonstrated that ASHMs may be inherited from epialleles of the parents or may be induced during development [7,8,28]. To determine the inheritance of histone modifications in the heterozygous status, we analyzed the correlation of allelic histone modifications between the F1 hybrids and their parents. First, based on the gene body-specific distribution patterns of the H3K27me3 and H3K36me3 modifications (Additional file 4), we quantitated the H3K27me3 and H3K36me3 modification levels by normalizing the reads as the number of reads per kilobase per million reads (RPKM) within the gene bodies. The correlation coefficients between the parent histone modification differences and the ASHM in the F1 hybrids ranged from 0.52 to 0.63 for both H3K27me3 and H3K36me3 (Figure 2A and D, blue spots, *P* < 0.001). A total of 463 H3K27me3-modified genes and 330 H3K36me3-modified genes were identified as differently modified genes between GL and 93–11 (fold change > 2, FDR < 0.05), 321 H3K27me3-modified genes and 330 H3K36me3-modified genes were identified as differently modified genes between GL and TQ. These differentially modified genes between the parents were designated as epialleles. Interestingly, strong correlation coefficients were detected for the epialleles (from 0.79 to 0.86) (Figure 2A and D, red spots). Furthermore, 52.48% (243/463 genes) and 70.40% (226/321 genes) of the H3K27me3 epialleles in the GL versus 93–11 and 53.33% (176/330 genes) and 54.41% (142/261 genes) of the H3K36me3 epialleles (176 and 142 genes) in GL versus TQ were identified as ASHM in their F1 hybrids (Figure 2E). These results indicate that epialleles mainly contribute to ASHM in F1 hybrids.

### Inheritance and reprogramming of H3K27me3 and H3K36me3 in F1 hybrids exhibited biological functional diversity

**Figure 3:**
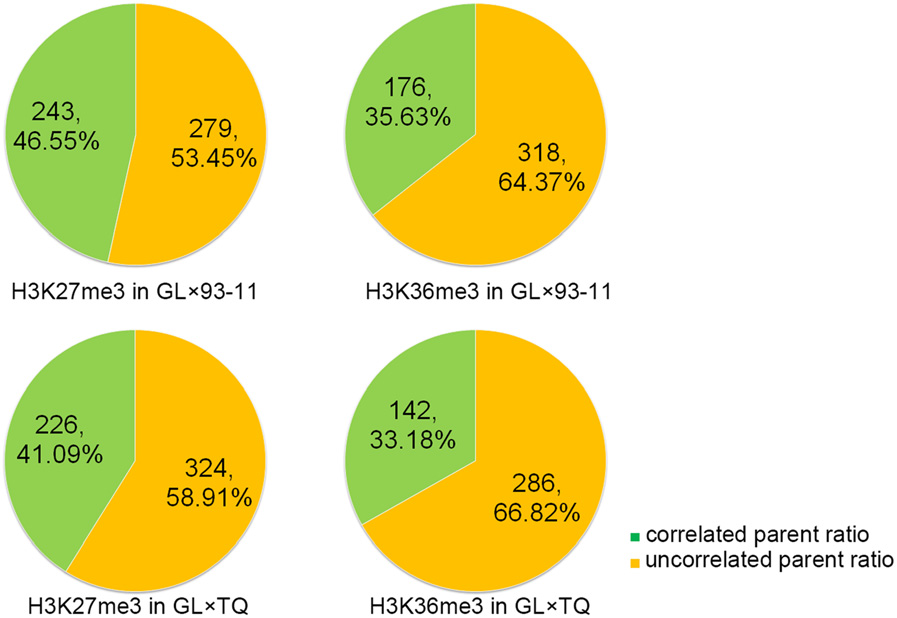
**The parent modification ratio of the ASHM genes.** Orange represents the ASHM genes that exhibited the same histone modification differences between the parents; green represents the ASHM genes with equal histone or uncorrelated modification between the parents.

**Figure 4:**
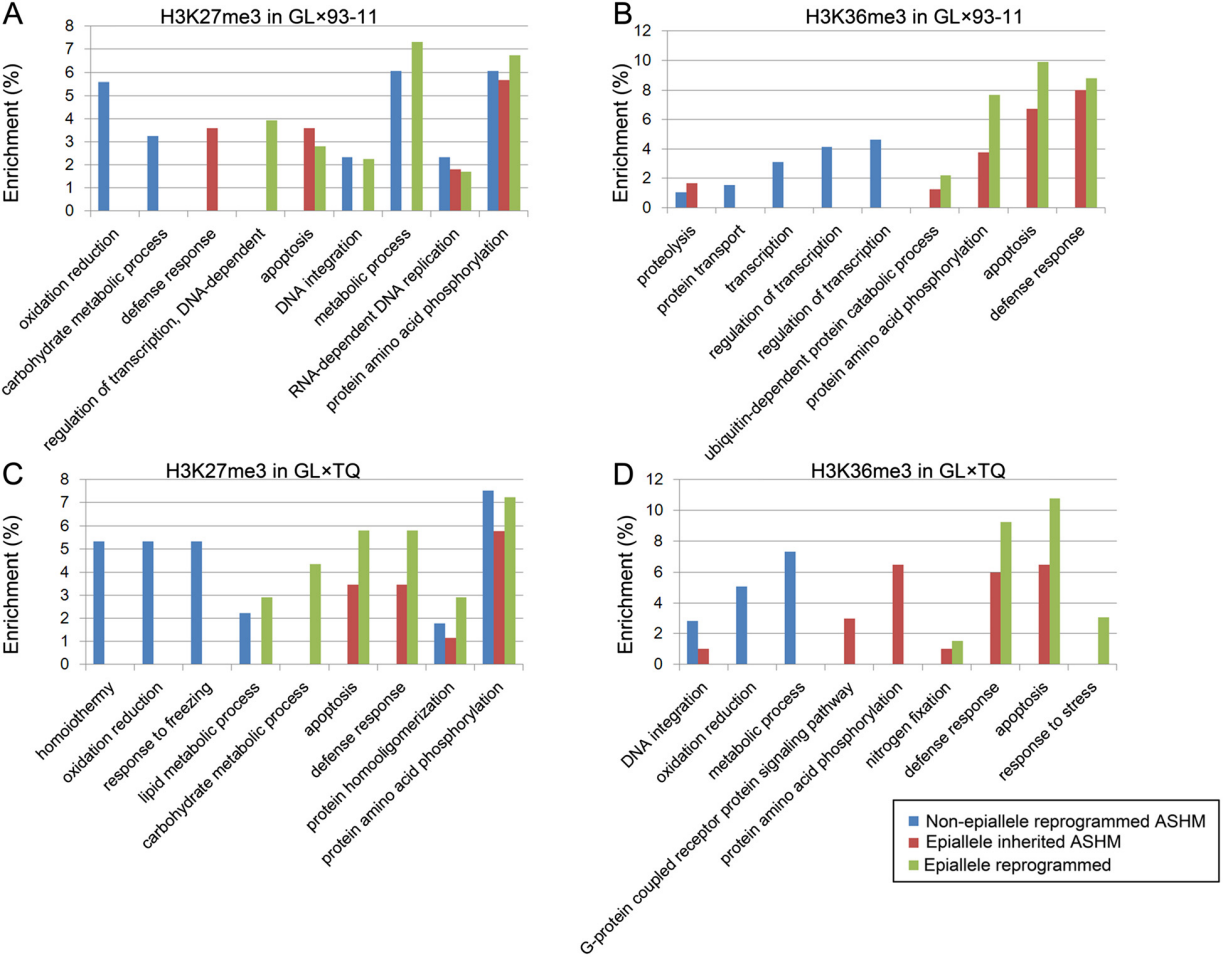
**Gene ontology classification of the genes with different epigenetic inheritance patterns in each F1 hybrid. (A-D)**, Gene ontology classification of H3K27me3 or H3K36me3 modified genes with different epigenetic inheritance patterns in GL × 93-11 or GL × TQ.

In addition to epiallele inheritance from parents, alternative processes could generate allelic histone modifications resulting from the reprogramming of histone modifications in F1 hybrids [7,8,28]. To investigate the epiallele inheritance and reprogramming of H3K27me3 and H3K36me3 from parents to F1 hybrids, we compared two histone allele-specific modification differences between both of the parents in the F1 hybrids. The results showed that 53.45% (279/522) to 58.91% (324/550) of the allele-specific H3K27me3 genes and 64.37% (318/494) to 66.82% (286/428) of the allele-specific H3K36me3 genes were modified in the F1 hybrids. However, the histone modification levels of these genes did not show differences between the parents (Figure 3). The reprogramming of the ASHM genes occurred in the F1 hybrid. Therefore, we speculate that the inheritance and reprogramming of the ASHM genes could present functional diversity in the F1 hybrids. To confirm this hypothesis, we performed a gene ontology enrichment analysis. These results demonstrate that inherited epialleles with either H3K27me3 or H3K36me3 enrichment were involved in the biological processes of apoptosis and defense response (Figure 4, red portion), whereas the reprogrammed ASHM genes were involved in the regulation of transcription, metabolic processes, oxidation-reduction and proteolysis (Figure 4, blue portion). In addition, the reprogramming of biallelic histone modification (BAHM) or ASHM genes in the F1 hybrids exhibited functional diversity (Figure 4, green portion). Our data indicate clear functional diversity between the inheritance and reprogramming of the ASHM genes in the F1 hybrid, which could help elucidate the regulatory mechanisms of the two types of allelic histone modifications for gene expression profiles.

### Differentially modified genes between parents and hybrids are attributed to ASHM genes in the F1 hybrids

**Figure 5:**
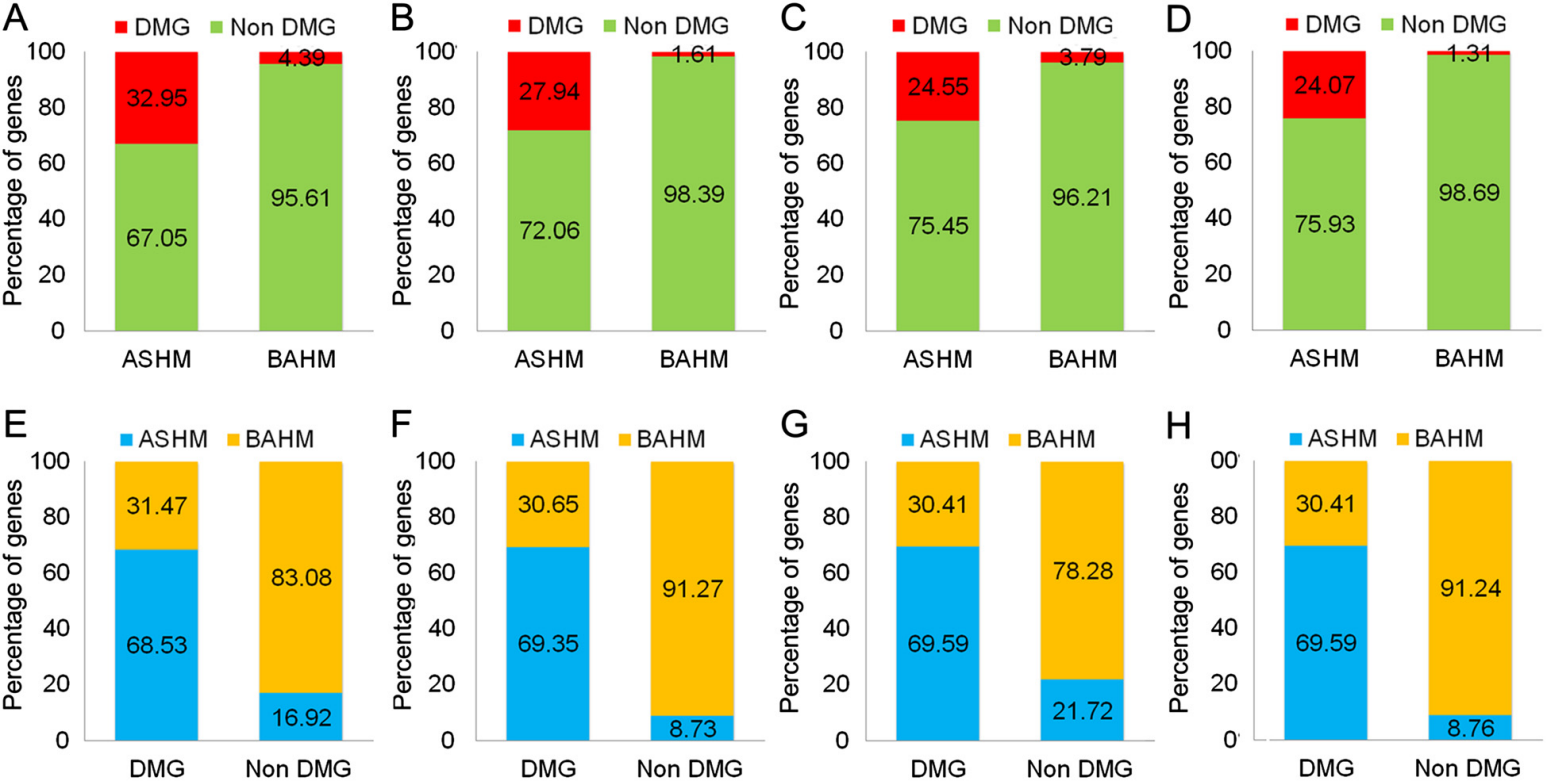
**Relationship between differentially modified genes between hybrids, parents and ASHM in GL × 93-11 and GL × TQ. (A-D)**, The percentage of DMGs and non-DMGs in ASHM and BAHM; **(E-H)**, the percentage of ASHM genes and BAHM genes in DMGs and non-DMGs.

To determine whether ASHMs contribute to differentially modified genes, we analyzed the RPKM level of the ASHM genes. Of the analyzed genes, 11.09% to 26.13% of the genes were ASHM genes with either H3K27me3 or H3K36me3 modifications (Figure 1). To further explore whether ASHM genes could lead to differences in histone modification, we analyzed the differential modification genes of H3K27me3 or H3K36me3 modifications between the F1 hybrid and the parents. We found that 24.07% to 32.95% of the ASHM genes with H3K27me3 or H3K36me3 modifications contributed to 68.53% to 69.59% of the differentially modified genes between the parents and the hybrids (Figure 5) In contrast, only 8.73% to 21.72% of the non-differentially modified genes exhibited ASHM genes (Figure 5E and H). Taken together, our results indicate that ASHMs primarily contribute to differentially modified genes in F1 hybrids.

### ASHM-H3K36me3 involved in regulating allelic specific gene expression

**Figure 6:**
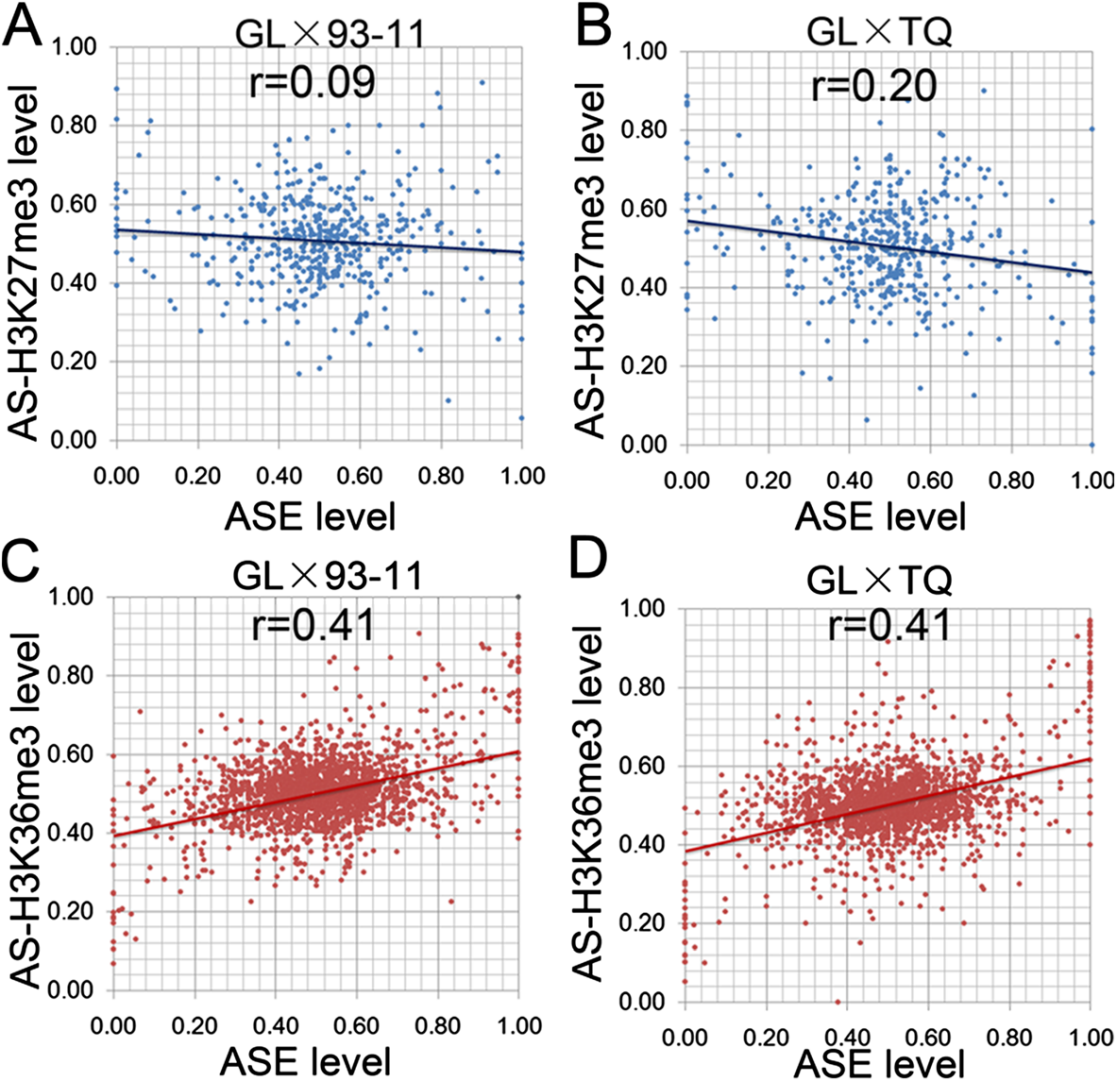
**Correlation of histone modifications and ASHM between the parents and the F1 hybrid. (A and B)**, The correlation between H3K27me3-ASHM (Y axis) and the ASE level (X axis) from the GL alleles in GL × 93-11 and GL × TQ. **(C and D**), The correlation between H3K36me3-ASHM (Y axis) and the ASE level (X axis) from the GL alleles in GL × 93-11 and GL × TQ. The ASE and ASHM levels are presented as the ratio of GL expression or modification level to the total expression or histone modification level of the two alleles in the F1 hybrid, respectively.

Previous studies have demonstrated that ASHMs regulate allele-specific expression (ASE) in mice [25]. To investigate the effect of ASHMs on ASE in rice F1 hybrids, we analyzed the relationship between the expression of allele-specific genes and allele-specific modifications in the two F1 hybrids. The ASE genes that were detected in GL × 93-11 and GL × TQ were used for this study [27]. We found that 436 to 478 of the ASE genes exhibited H3K27me3 modification and that 1787 to 1,973 of the ASE genes exhibited H3K36me3 modification in both of the F1 hybrids. No significant correlation of the allelic H3K27me3 with ASE (r = 0.09, *p* < 0.001, r = 0.20, *p* < 0.001) was detected in either of the F1 hybrids (Figure 6A and B). However, a positive correlation of the allelic H3K36me3 with ASE was detected in both of the F1 hybrids (r = 0.41, *p* < 0.001) (Figure 6C and D). These results indicate that H3K36me3 is involved in the regulation of ASE, whereas H3K27me3 is not. Our data indicate that different histone modifications play different roles in regulating ASE.

**Figure 7:**
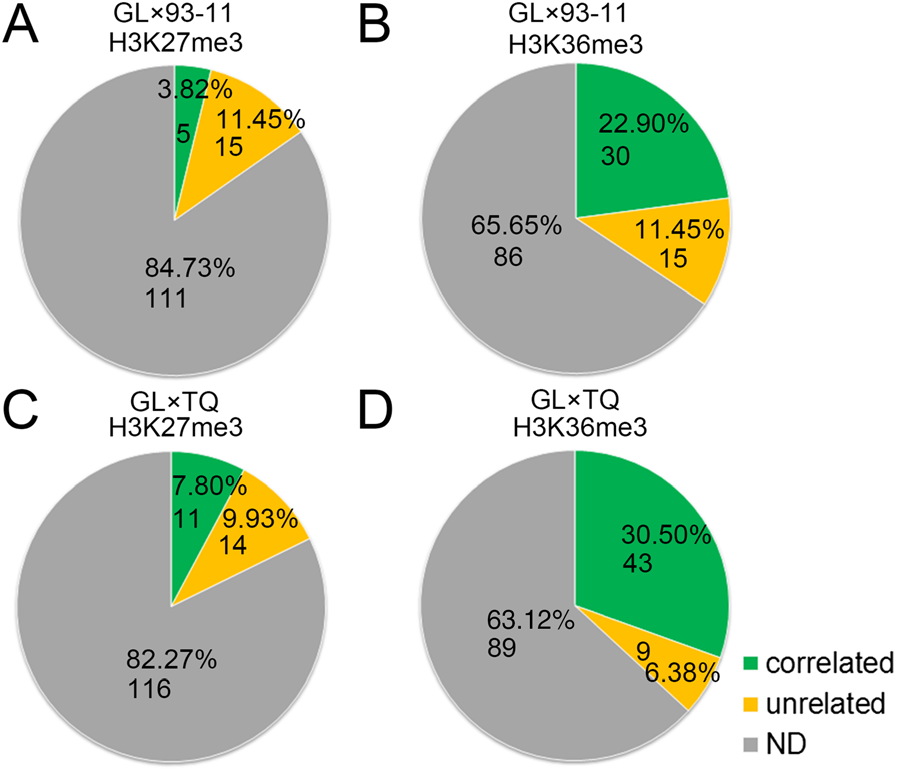
**The ratio of allelic histone modification of monoallelic expression genes in rice F1 hybrids. (A-D)**, The ratio of allelic H3K27me3 or H3K36me3 of monoallelic expression genes in GL × 93-11 or GL × TQ.

Monoallelic expression genes are thought to be extremely important for development [20-22]. However, the roles of epigenetic elements in monoallelic expression genes are not fully understood, especially in higher plants. In previous study, 129 and 143 monoallelic expression genes were identified in GL × 93-11 and GL × TQ, respectively, and no imprinted genes have been found [27]. A total of 65 monoallelic expression genes in GL × 93-11 and 77 monoallelic expression genes with histone modifications in GL × TQ were detected. We found that 30 of 45 (66.67%) monoallelic expression genes exhibited ASHM-H3K36me3, and five of 20 monoallelic expression genes exhibited the H3K27me3 modification in GL × 93-11 (Figure 7A and B, Additional files 5 and 6). The same results were observed in GL × TQ; i.e., 11 (44.00%) of 25 monoallelic expression genes exhibited ASHM-H3K27me3 (Figure 7C, Additional file 7), whereas 43 (82.69%) of 52 monoallelic expression genes exhibited ASHM-H3K36me3 (Figure 7D, Additional file 8). These results indicate that H3K36me3 primarily contributes to monoallelic expression in the rice F1 hybrids.

## Discussion

Although global epigenetic modifications have been investigated in *japonica*-*indica* hybrids of Nipponbare and 93–11 [11,26], a study focusing on *indica-indica* hybrids in foundation varieties has not been conducted. In this study, we studied global allele-specific epigenetic modifications by performing high-depth ChIP-seq of H3K27me3 and H3K36me3 in two *indica-indica* F1 hybrids, GL/93-11 and GL/TQ. Our epigenome data with two histone modifications demonstrate that ASHMs are widely detected in F1 hybrids. Our data will be helpful in understanding the relationships between allelic histone modifications and ASE in rice F1 hybrids.

More than half of the ASHM genes in the F1 hybrids were equally modified in the parents (Figure 2E), and the epialleles were inherited and reprogrammed in the F1 hybrids. We detected a large number of epialleles, and those that contributed to ASHM caused a large number of differentially modified genes in the F1 hybrids. These results indicate that epialleles play important roles in ASHM and in differentially modified genes. Furthermore, 52.48% to 70.40% of epialleles were inherited by F1 hybrids, and 29.60% to 47.52% of the epialleles were reprogrammed in the F1 hybrids (Figure 2E). More interestingly, the inheritance and reprogramming of epialleles were involved in different biological processes (Figure 4). The reprogramming of the epialleles in the F1 hybrid, derived from genetic polymorphism of SNPs, provided more options to regulate the global gene expression profile in the F1 hybrid. Thus, the reprogramming of histone modifications between alleles in the F1 hybrids may cause superior fitness under various environmental conditions, including biotic and non-biotic stresses, which may contribute to heterosis.

Although histone modifications are generally recognized as epigenetic modifications, not all histone modifications are heritable [29]. However, histone methylation is quite stable compared to other modifications, such as histone phosphorylation and acetylation, during the cell cycle. We found strong correlations between the epialleles and ASHM (Figure 2A and D). The majority of differentially modified genes were derived from ASHM genes (Figure 5E and H). Previous studies have demonstrated that differentially modified genes are closely related to differentially expressed genes [11], which are widely recognized as the basis of heterosis [30]. Our findings of the relationships between ASHM, differentially modified genes and differentially expressed genes in F1 hybrids indicate that the epialleles could provide a genetic basis for heterosis and general combining ability [31]. Therefore, epialleles may be used as criteria for screening hybrids with higher heterosis in hybrid rice breeding programs.

In general, histone methylations that are associated with gene silencing (e.g., H3K27me3) are more stable than are histone methylations that are associated with gene activation (e.g., H3K36me3) [32]. In this study, 22.50% to 26.13% and 11.09% to 11.56% of the analyzed genes exhibited ASHM in H3K27me3 and H3K36me3 modifications, respectively. The proportions of allele-specific H3K27me3 were much greater than were those of allele-specific H3K36me3 in both of the F1 hybrids (Figure 1), although the ASHM gene numbers were approximately equal, indicating that H3K36me3 modification is more effective in ASHM than H3K27me3 modification. Our data indicate that the regulatory effects of H3K36me3 modification on ASE are greater than are those of the H3K27me3 modification, suggesting that different histone modifications may have different roles in the regulation of gene expression in F1 hybrids [31].

Several types of epigenetic modifications are involved in ASE regulation [17,25,33,34]. In mice, ASE or H3K4me3 enrichment (20 genes (21.3%)) are negatively correlated with allele-specific DNA methylation [25]. In *indica-japonica* hybrid rice, only 15 ASE genes (17.6%) have been reported to undergo allele-specific modification [26]. In this study, significant correlations were observed between allelic gene expression and allelic H3K36me3 but not allelic H3K27me3 (Figures 6 and 7). The histone modification frequency within the gene body is higher than that of DNA methylation in rice [11]. In contrast to DNA methylation and H3K27me3 modification, we found that H3K36me3 modification primarily regulates ASE. Our results indicate that ASHM-H3K36me3 mainly contributes to ASE, suggesting that ASHM-H3K36me3 could play a more important role in ASE than ASHM-H3K27me3 modifications.

## Conclusions

The profiles of H3K27me3 and H3K36me3 in two *indica-indica* F1 hybrids were investigated using ChIP-sequencing technology. A total of 522 to 550 allele-specific H3K27me3 genes and 428 to 494 allele-specific H3K36me3 genes were detected in GL × 93-11 and GL × TQ, accounting for 11.09% and 26.13% of the total analyzed genes, respectively. The epialleles between parents were highly related to ASHMs. ASHM genes mainly showed differential modification between parents and hybrids. Our findings indicate that ASHM-H3K36me3, rather than H3K27me3, mainly regulates ASE in hybrid rice.

## Methods

### Plant materials

Three rice *indica* varieties, Guangluai-4 (GL), Yangdao-6 (93–11) and Teqing (TQ), and two F1 hybrids, GL × 93-11 and GL × TQ, were grown in the summer of 2010 in Wuhan. The second fully expanded leaves were harvested at the secondary branch differentiation stage, immediately frozen in liquid nitrogen and stored at −80°C until use. The leaves from triplicate plots were pooled for ChIP.

### ChIP-Seq library generation

Chromatin immunoprecipitation (ChIP) was performed using antibodies against H3K27me3 (Abcam, Cat. #ab6002, Cambridge, MA 02139–1517, USA) and H3K36me3 (Abcam, Cat. #ab9050). DNA was extracted using an equal volume phenol:chloroform:isoamyl alcohol and briefly vortexed; DNA was precipitated with a 2.5-volume of 100% EtOH containing 0.3 M sodium acetate and 2 μl of glycogen (20 mg ml–1) at a pH of 5.2. Specificity of immunoprecipitation was verified by qPCR using the primers derived from *actin* and *copia* genes and the enrichment of immunoprecipitation was confirmed through comparing with input chromatin (Additional files 9 and 10). The resulting ChIP DNA was used to generate Illumina sequencing libraries according to the manufacturer’s protocol: suitable fragments of approximately 200 bp were selected as templates for amplification after incubation at 98°C for 30 s for denaturation, followed by 15 cycles of 98°C for 10 s, 65°C for 30 s, 72°C for 30 s and 72°C for 5 min. The samples were purified using the QIAquick PCR purification kit (Qiagen, Valencia,CA USA) as described in the manufacturer’s protocol. One microliter of the library was loaded on an Agilent Technologies 2100 Bioanalyzer using the Agilent DNA 1000 chip kit (Agilent, part #5067-1504). After verifying the DNA size and purity, the library was sequenced using the Illumina GAIIx platform by BGI in Shenzhen, China.

### Sequencing read alignment

Raw reads were filtered prior to data analysis, including the reads that contained only adaptor sequences, reads with more than 10% unknown bases and reads with more than half of the bases with a quality score of less than 5.0. After obtaining the clean reads, SOAP2 was used to map the reads to reference genome sequences from Nipponbare (http://www.gramene.org/) [35]. Only two mismatches were allowed in the alignment. Peak calling analysis was performed using Model-based Analysis of ChIP-Seq (MACS) software 1.4.0 (http://liulab.dfci.harvard.edu/MACS/00README.html) with default parameters (bandwidth, 300 bp; mfold, 32; *p*-value of 1.00e-05) to call peaks that represent enriched histone modifications [36] (Additional file 11). The number of reads per kilobase per million reads (RPKM) method was used to calculate the modification levels of unique genes (BGI, Shenzhen, China). We normalized the ChIP read counts by computing the number of RPKMs in the gene body region based on the gene body-specific distribution of both H3K27me3 and H3K36me3 as previously described [11,12,37]. The *P*-values and the FDRs of differentially histone modified genes were calculated as described [38]. The allele-specific histone methylation (ASHM) in the F1 hybrids was distinguished based on SNPs.

### Determination of ASHM

The ASHM genes were determined based on the reads in the gene body region. Briefly, more than nine reads in the gene body region were calculated as described by Song et al. [27]. The ASHMs that were derived from the paternal or maternal alleles were calculated by dividing the reads of each allele by the total number of reads. Three types of ASHMs were categorized: i.e. when only one allele of a gene was detected as modified, the gene was categorized as a monoallelic modified gene; when the modification level of an allele was 2-fold greater than that of another allele, the gene was categorized as an allele-specific histone modification; and when the modification level of an allele was biased to one parent by less than 2-fold, the gene was categorized as a biallelic histone-modified gene.

### GO and statistical analyses

GO analysis was performed using the open-source Rice Oligonucleotide Array Database (ROAD) (http://www.ricearray.org/analysis/go_enrichment.shtml) [39]. The *t*-test and correlation analysis were conducted using Microsoft Office Excel 2010.

## Availability of supporting data

The ChIP-seq reported in this paper has been deposited in the Gene Expression Omnibus (GEO) database, www.ncbi.nlm.nih.gov/geo (accession no. GSE66537).

The ASHM and RPKM data has been deposited in the LabArchives, https://mynotebook.labarchives.com/ (DOI: 10.6070/H4JW8BVR).

## Additional files

Additional file 1:
**Illumina sequencing of DNA from the chromatin immunoprecipitation (ChIP-seq) of H3K27me3. **
Additional file 2:
**Illumina sequencing of DNA from the chromatin immunoprecipitation (ChIP-seq) of H3K36me3.**
Additional file 3:
**Read number of each allele in F1.**
Additional file 4:
**Distribution of H3K27me3 and H3K36me3 modification levels within the gene region.**
Additional file 5:
**Allele-specific H3K27me3 modification of monoallelic expression genes in GL × 93-11.**
Additional file 6:
**Allele-specific H3K36me3 modification of monoallelic expression genes in GL × 93-11.**
Additional file 7:
**Allele-specific H3K27me3 modification of monoallelic expression genes in GL × TQ.**
Additional file 8:
**Allele-specific H3K36me3 modification of monoallelic expression genes in GL × TQ.**
Additional file 9:
**Specificity and enrichment of H3K27me3 ChIP.**
Additional file 10:
**Specificity and enrichment of H3K36me3 ChIP.**
Additional file 11:
**Peaks number of H3K27me3 and H3K36me3.**

## Abbreviations

ASHM: Aallele-specific histone modification
ASE: Allele-specific gene expression
BAHM: Biallelic histone modification
ChIP-seq: Chromatin immunoprecipitation followed by next-generation sequencing
DMGs: Differentially modified genes between parents and hybrid
FDR: False discovery rate
H3K27me3: Trimethylation on histone H3 lysine 27
H3K36me3: Trimethylation histone H3 lysine 36
RPKM: Reads per kilobase per million reads
