# Supplementary material for: Global epigenomic analysis indicates that Epialleles contribute to Allele-specific expression via Allele-specific histone modifications in hybrid rice
Source: BMC Genomics. 2015 Mar 24;16(1):232. doi: 10.1186/s12864-015-1454-z (PMC4394419; doi:10.1186/s12864-015-1454-z)
Supplement: Additional file 1: — Illumina sequencing of DNA from the chromatin immunoprecipitation (ChIP-seq) of H3K27me3. [file 12864_2015_1454_MOESM1_ESM.doc]

Additional file 1. The Illumina sequencing of DNA from chromatin immunoprecipitation (ChIP-seq) of H3K27me3

|  | clean reads number | Unique mapped reads | Unique mapped rates |
| --- | --- | --- | --- |
| GL | 60,751,176 | 42,235,944 | 69.52% |
| GL×93-11 | 61,042,282 | 43,755,981 | 71.68% |
| GL×TQ | 58,658,906 | 42,531,652 | 72.51% |
| 93-11 | 58,093,668 | 42,879,270 | 73.81% |
| TQ | 50,639,787 | 36,356,272 | 71.79% |
