# Supplementary material for: Global epigenomic analysis indicates that Epialleles contribute to Allele-specific expression via Allele-specific histone modifications in hybrid rice
Source: BMC Genomics. 2015 Mar 24;16(1):232. doi: 10.1186/s12864-015-1454-z (PMC4394419; doi:10.1186/s12864-015-1454-z)
Supplement: Additional file 2: — Illumina sequencing of DNA from the chromatin immunoprecipitation (ChIP-seq) of H3K36me3. [file 12864_2015_1454_MOESM2_ESM.doc]

Additional file 2. The Illumina sequencing of DNA from chromatin immunoprecipitation (ChIP-seq) of H3K36me3

|  | clean reads number | Unique mapped reads | Unique mapped rates |
| --- | --- | --- | --- |
| GL | 62,173,975 | 47,720,307 | 76.75% |
| GL×93-11 | 56,887,926 | 43,411,168 | 76.31% |
| GL×TQ | 58,349,263 | 44,982,237 | 77.09% |
| 93-11 | 48,917,838 | 37,305,130 | 76.26% |
| TQ | 64,097,715 | 49,819,855 | 77.72% |
