# Supplementary material for: Global epigenomic analysis indicates that Epialleles contribute to Allele-specific expression via Allele-specific histone modifications in hybrid rice
Source: BMC Genomics. 2015 Mar 24;16(1):232. doi: 10.1186/s12864-015-1454-z (PMC4394419; doi:10.1186/s12864-015-1454-z)
Supplement: Additional file 3: — Read number of each allele in F1. [file 12864_2015_1454_MOESM3_ESM.doc]

Additional file 3. Reads number of each allele in F1

|  | GL×93-11 | | GL×TQ | |
| --- | --- | --- | --- | --- |
|  | GL allele | 93-11 allele | GL allele | TQ allele |
| H3K36me3 reads number | 1，071，381 | 1，041，100 | 1，036，821 | 1，022，242 |
| H3K27me3 reads number | 1，542，303 | 1，441，134 | 1，436，238 | 1，405，011 |
