# Supplementary figures and images for: Global epigenomic analysis indicates that Epialleles contribute to Allele-specific expression via Allele-specific histone modifications in hybrid rice
Source: BMC Genomics. 2015 Mar 24;16(1):232. doi: 10.1186/s12864-015-1454-z (PMC4394419; doi:10.1186/s12864-015-1454-z)

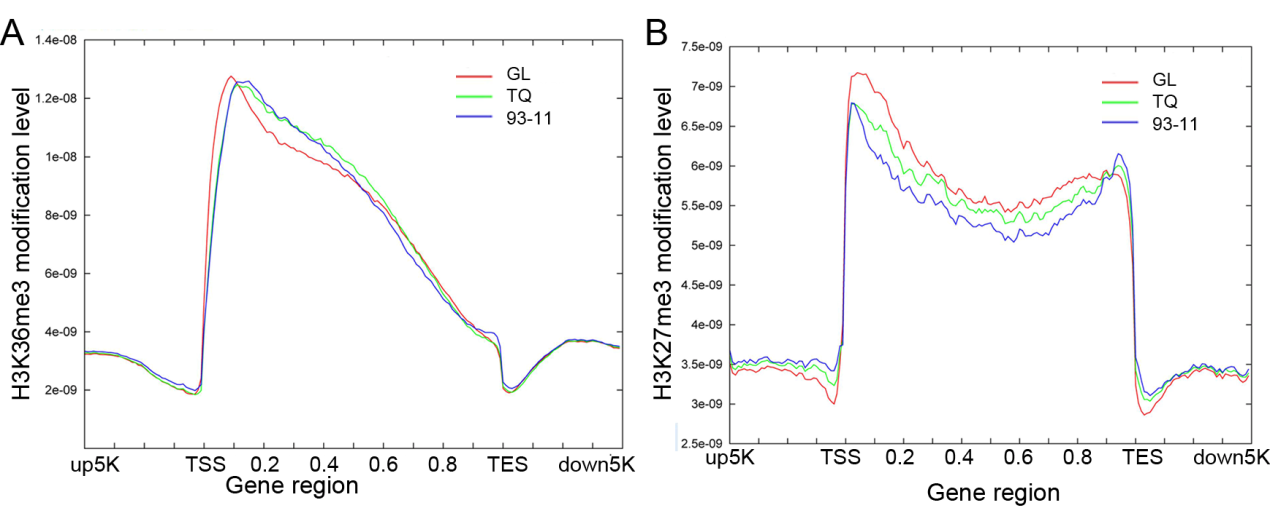


Additional file 4. Distribution of H3K27me3 and H3K36me3 modification levels within gene region.

Supplement: Additional file 4: — Distribution of H3K27me3 and H3K36me3 modification levels within the gene region. [file 12864_2015_1454_MOESM4_ESM.doc]
