# Supplementary material for: Global epigenomic analysis indicates that Epialleles contribute to Allele-specific expression via Allele-specific histone modifications in hybrid rice
Source: BMC Genomics. 2015 Mar 24;16(1):232. doi: 10.1186/s12864-015-1454-z (PMC4394419; doi:10.1186/s12864-015-1454-z)
Supplement: Additional file 5: — Allele-specific H3K27me3 modification of monoallelic expression genes in GL × 93-11. [file 12864_2015_1454_MOESM5_ESM.doc]

Additional file 5. Allele-specific H3K27me3 modification of mono-allelic expression genes in GL×93-11

| Gene | ASE level of GL allele | allelic H3K27me3 level of GL allele |
| --- | --- | --- |
| LOC_Os01g33960 | 0.00 | 0.52 |
| LOC_Os01g42330 | 0.00 | 0.39 |
| LOC_Os04g41000 | 0.00 | 0.56 |
| LOC_Os04g52590 | 0.00 | 0.64 |
| LOC_Os06g39090 | 0.00 | 0.59 |
| LOC_Os07g04480 | 0.00 | 0.82 |
| LOC_Os07g10940 | 0.00 | 0.54 |
| LOC_Os08g10250 | 0.00 | 0.65 |
| LOC_Os08g21879 | 0.00 | 0.48 |
| LOC_Os11g15450 | 0.00 | 0.89 |
| LOC_Os11g29090 | 0.00 | 0.62 |
| LOC_Os11g40009 | 0.00 | 0.53 |
| LOC_Os02g05530 | 1.00 | 0.46 |
| LOC_Os07g45560 | 1.00 | 0.34 |
| LOC_Os10g04730 | 1.00 | 0.40 |
| LOC_Os10g24000 | 1.00 | 0.06 |
| LOC_Os11g41210 | 1.00 | 0.33 |
| LOC_Os11g41540 | 1.00 | 0.50 |
| LOC_Os11g45190 | 1.00 | 0.26 |
| LOC_Os12g24800 | 1.00 | 0.48 |
