# Supplementary material for: Global epigenomic analysis indicates that Epialleles contribute to Allele-specific expression via Allele-specific histone modifications in hybrid rice
Source: BMC Genomics. 2015 Mar 24;16(1):232. doi: 10.1186/s12864-015-1454-z (PMC4394419; doi:10.1186/s12864-015-1454-z)
Supplement: Additional file 6: — Allele-specific H3K36me3 modification of monoallelic expression genes in GL × 93-11. [file 12864_2015_1454_MOESM6_ESM.doc]

Additional file 6. Allele-specific H3K36me3 modification of mono-allelic expression genes in GL×93-11

| Gene | ASE level | allelic H3K36me3 level |
| --- | --- | --- |
| LOC_Os01g09320 | 0.00 | 0.48 |
| LOC_Os01g20880 | 0.00 | 0.32 |
| LOC_Os01g33960 | 0.00 | 0.59 |
| LOC_Os03g32330 | 0.00 | 0.10 |
| LOC_Os04g41000 | 0.00 | 0.25 |
| LOC_Os04g52590 | 0.00 | 0.46 |
| LOC_Os06g38680 | 0.00 | 0.40 |
| LOC_Os06g39090 | 0.00 | 0.44 |
| LOC_Os07g04480 | 0.00 | 0.37 |
| LOC_Os07g04490 | 0.00 | 0.07 |
| LOC_Os07g30980 | 0.00 | 0.42 |
| LOC_Os08g14860 | 0.00 | 0.12 |
| LOC_Os08g18079 | 0.00 | 0.46 |
| LOC_Os08g21879 | 0.00 | 0.17 |
| LOC_Os08g27580 | 0.00 | 0.19 |
| LOC_Os10g04750 | 0.00 | 0.20 |
| LOC_Os11g44990 | 0.00 | 0.18 |
| LOC_Os12g20410 | 0.00 | 0.25 |
| LOC_Os01g14790 | 1.00 | 0.49 |
| LOC_Os01g32439 | 1.00 | 0.73 |
| LOC_Os01g55090 | 1.00 | 0.83 |
| LOC_Os01g56850 | 1.00 | 0.59 |
| LOC_Os02g05530 | 1.00 | 0.60 |
| LOC_Os02g31230 | 1.00 | 0.84 |
| LOC_Os03g01420 | 1.00 | 0.82 |
| LOC_Os04g22950 | 1.00 | 0.76 |
| LOC_Os04g23040 | 1.00 | 0.88 |
| LOC_Os04g23140 | 1.00 | 0.90 |
| LOC_Os04g30180 | 1.00 | 0.75 |
| LOC_Os04g38060 | 1.00 | 0.51 |
| LOC_Os05g08900 | 1.00 | 0.39 |
| LOC_Os06g42650 | 1.00 | 0.69 |
| LOC_Os07g01900 | 1.00 | 0.68 |
| LOC_Os07g45560 | 1.00 | 0.71 |
| LOC_Os08g14880 | 1.00 | 0.73 |
| LOC_Os08g41630 | 1.00 | 0.54 |
| LOC_Os10g03669 | 1.00 | 1.00 |
| LOC_Os10g04342 | 1.00 | 0.40 |
| LOC_Os10g15240 | 1.00 | 0.76 |
| LOC_Os11g27799 | 1.00 | 0.81 |
| LOC_Os11g39310 | 1.00 | 0.91 |
| LOC_Os11g41540 | 1.00 | 0.71 |
| LOC_Os12g13295 | 1.00 | 0.88 |
| LOC_Os12g22010 | 1.00 | 0.69 |
| LOC_Os12g24800 | 1.00 | 0.81 |
