# Supplementary material for: Global epigenomic analysis indicates that Epialleles contribute to Allele-specific expression via Allele-specific histone modifications in hybrid rice
Source: BMC Genomics. 2015 Mar 24;16(1):232. doi: 10.1186/s12864-015-1454-z (PMC4394419; doi:10.1186/s12864-015-1454-z)
Supplement: Additional file 7: — Allele-specific H3K27me3 modification of monoallelic expression genes in GL × TQ. [file 12864_2015_1454_MOESM7_ESM.doc]

Additional file 7. Allele-specific H3K27me3 modification of mono-allelic expression genes in GL×TQ

| Gene | ASE level of GL allele | allelic H3K27me3 level of GL allele |
| --- | --- | --- |
| LOC_Os04g23360 | 0.00 | 0.34 |
| LOC_Os04g52590 | 0.00 | 0.38 |
| LOC_Os05g13420 | 0.00 | 0.38 |
| LOC_Os05g43910 | 0.00 | 0.46 |
| LOC_Os05g48790 | 0.00 | 0.73 |
| LOC_Os07g04480 | 0.00 | 0.89 |
| LOC_Os07g10940 | 0.00 | 0.54 |
| LOC_Os09g19390 | 0.00 | 0.77 |
| LOC_Os10g25180 | 0.00 | 0.64 |
| LOC_Os11g01990 | 0.00 | 0.86 |
| LOC_Os11g40009 | 0.00 | 0.60 |
| LOC_Os11g47140 | 0.00 | 0.63 |
| LOC_Os12g36030 | 0.00 | 0.87 |
| LOC_Os01g10700 | 1.00 | 0.34 |
| LOC_Os03g13690 | 1.00 | 0.31 |
| LOC_Os03g57560 | 1.00 | 0.57 |
| LOC_Os04g30030 | 1.00 | 0.38 |
| LOC_Os07g19210 | 1.00 | 0.32 |
| LOC_Os08g01520 | 1.00 | 0.41 |
| LOC_Os09g19229 | 1.00 | 0.80 |
| LOC_Os10g24000 | 1.00 | 0.00 |
| LOC_Os10g24050 | 1.00 | 0.25 |
| LOC_Os11g41210 | 1.00 | 0.18 |
| LOC_Os11g45190 | 1.00 | 0.23 |
| LOC_Os11g45990 | 1.00 | 0.37 |
