# Supplementary material for: Global epigenomic analysis indicates that Epialleles contribute to Allele-specific expression via Allele-specific histone modifications in hybrid rice
Source: BMC Genomics. 2015 Mar 24;16(1):232. doi: 10.1186/s12864-015-1454-z (PMC4394419; doi:10.1186/s12864-015-1454-z)
Supplement: Additional file 8: — Allele-specific H3K36me3 modification of monoallelic expression genes in GL × TQ. [file 12864_2015_1454_MOESM8_ESM.doc]

Additional file 8. Allele-specific H3K36me3 modification of mono-allelic expression genes in GL×TQ

| Gene | ASE level of GL allele | allelic H3K36me3 level of GL allele |
| --- | --- | --- |
| LOC_Os02g14520 | 0.00 | 0.41 |
| LOC_Os04g51009 | 0.00 | 0.77 |
| LOC_Os04g52590 | 0.00 | 0.38 |
| LOC_Os05g13420 | 0.00 | 0.38 |
| LOC_Os05g48790 | 0.00 | 0.73 |
| LOC_Os06g07810 | 0.00 | 0.59 |
| LOC_Os06g12470 | 0.00 | 0.57 |
| LOC_Os06g38680 | 0.00 | 0.85 |
| LOC_Os06g39090 | 0.00 | 0.43 |
| LOC_Os07g09460 | 0.00 | 0.47 |
| LOC_Os10g04750 | 0.00 | 0.60 |
| LOC_Os11g01990 | 0.00 | 0.86 |
| LOC_Os11g08940 | 0.00 | 0.55 |
| LOC_Os11g44960 | 0.00 | 0.43 |
| LOC_Os11g44990 | 0.00 | 0.50 |
| LOC_Os12g02060 | 0.00 | 0.57 |
| LOC_Os12g02070 | 0.00 | 0.56 |
| LOC_Os12g20410 | 0.00 | 0.31 |
| LOC_Os12g28100 | 0.00 | 0.85 |
| LOC_Os12g32310 | 0.00 | 0.33 |
| LOC_Os02g05530 | 1.00 | 0.59 |
| LOC_Os02g18612 | 1.00 | 0.00 |
| LOC_Os02g31230 | 1.00 | 0.64 |
| LOC_Os02g38386 | 1.00 | 0.29 |
| LOC_Os02g38392 | 1.00 | 0.49 |
| LOC_Os03g01420 | 1.00 | 0.43 |
| LOC_Os03g01520 | 1.00 | 0.62 |
| LOC_Os03g13690 | 1.00 | 0.31 |
| LOC_Os03g14920 | 1.00 | 0.29 |
| LOC_Os03g57560 | 1.00 | 0.57 |
| LOC_Os04g12990 | 1.00 | 0.45 |
| LOC_Os04g23040 | 1.00 | 0.41 |
| LOC_Os04g30180 | 1.00 | 0.70 |
| LOC_Os04g51250 | 1.00 | 0.50 |
| LOC_Os04g54110 | 1.00 | 0.57 |
| LOC_Os05g03320 | 1.00 | 0.49 |
| LOC_Os05g46660 | 1.00 | 0.50 |
| LOC_Os06g12140 | 1.00 | 0.60 |
| LOC_Os07g01890 | 1.00 | 0.50 |
| LOC_Os07g01900 | 1.00 | 0.58 |
| LOC_Os07g07030 | 1.00 | 0.75 |
| LOC_Os07g17689 | 1.00 | 0.44 |
| LOC_Os07g47110 | 1.00 | 0.58 |
| LOC_Os09g15639 | 1.00 | 0.77 |
| LOC_Os09g24170 | 1.00 | 0.45 |
| LOC_Os10g15240 | 1.00 | 0.89 |
| LOC_Os11g13680 | 1.00 | 0.11 |
| LOC_Os11g27799 | 1.00 | 0.67 |
| LOC_Os11g39190 | 1.00 | 0.86 |
| LOC_Os12g24800 | 1.00 | 0.29 |
| LOC_Os12g28110 | 1.00 | 0.36 |
| LOC_Os12g30760 | 1.00 | 0.49 |
