# Supplementary material for: Global epigenomic analysis indicates that Epialleles contribute to Allele-specific expression via Allele-specific histone modifications in hybrid rice
Source: BMC Genomics. 2015 Mar 24;16(1):232. doi: 10.1186/s12864-015-1454-z (PMC4394419; doi:10.1186/s12864-015-1454-z)
Supplement: Additional file 9: — Specificity and enrichment of H3K27me3 ChIP. [file 12864_2015_1454_MOESM9_ESM.doc]

Additional file 9 Specificity and enrichment of H3K27me3 ChIP

| H3K27me3 ChIP | △Ct | enrichment | primer |
| --- | --- | --- | --- |
| GL | 3.91 | 15 | CQ-6 |
| GL×93-11 | 2.81 | 7 | CQ-6 |
| GL×TQ | 3.93 | 15.2 | CQ-6 |
| 93-11 | 4.16 | 17.9 | CQ-6 |
| TQ | 3.64 | 12.4 | CQ-6 |

Primer CQ-6 was used to detect the enrichment H3K27me3 IP by BGI. No enrichment was detected using actin primer (actinF 5'- CGTGGGTGGGACTGAGAAGC -3', actinR 5'- TGGCGAGGTTGGTAGGTAGGTTAC -3' ).
