# Supplementary material for: Global epigenomic analysis indicates that Epialleles contribute to Allele-specific expression via Allele-specific histone modifications in hybrid rice
Source: BMC Genomics. 2015 Mar 24;16(1):232. doi: 10.1186/s12864-015-1454-z (PMC4394419; doi:10.1186/s12864-015-1454-z)
Supplement: Additional file 10: — Specificity and enrichment of H3K36me3 ChIP. [file 12864_2015_1454_MOESM10_ESM.doc]

Additional file 10 Specificity and enrichment of H3K36me3 ChIP

| H3K36me3 ChIP | △Ct | enrichment | primer |
| --- | --- | --- | --- |
| GL | 2.52 | 5.7 | actin |
| GL×93-11 | 2.32 | 5 | actin |
| GL×TQ | 2.47 | 5.5 | actin |
| 93-11 | 2.21 | 4.6 | actin |
| TQ | 2.64 | 6.2 | actin |

Primer actin was used to detect the enrichment H3K27me3 IP by BGI. No enrichment was detected using copia primer (copiaF, 5'- TGGCAACAAAAGAGTTCGACG -3', copiaR, 5'- TCCCCATCATTGGGTTCCTC -3').
