# Supplementary material for: Global epigenomic analysis indicates that Epialleles contribute to Allele-specific expression via Allele-specific histone modifications in hybrid rice
Source: BMC Genomics. 2015 Mar 24;16(1):232. doi: 10.1186/s12864-015-1454-z (PMC4394419; doi:10.1186/s12864-015-1454-z)
Supplement: Additional file 11: — Peaks number of H3K27me3 and H3K36me3. [file 12864_2015_1454_MOESM11_ESM.doc]

Additional file 11. Peaks number of H3K27me3 and H3K36me3

|  | H3K27me3 Peak number | H3K36me3 Peak number |
| --- | --- | --- |
| GL | 9,639 | 9,738 |
| GL×93-11 | 8,392 | 10,136 |
| GL×TQ | 8,621 | 9,505 |
| 93-11 | 9,054 | 9,714 |
| TQ | 8,385 | 9,870 |
